# Novel *Vibrio* spp. Strains Producing Omega-3 Fatty Acids Isolated from Coastal Seawater

**DOI:** 10.3390/md18020099

**Published:** 2020-02-01

**Authors:** Mónica Estupiñán, Igor Hernández, Eduardo Saitua, M. Elisabete Bilbao, Iñaki Mendibil, Jorge Ferrer, Laura Alonso-Sáez

**Affiliations:** 1 AZTI, Marine Research Division, Txatxarramendi Irla s/n, 48395 Sukarrieta, Spain; mestupinan@azti.es (M.E.); ebilbao@azti.es (M.E.B.); imendibil@azti.es (I.M.); 2 AZTI, Food Research Division, Astondo Bidea, Building 609, 48160 Derio, Spain; igor.hernandezo@ehu.eus (I.H.); esaitua@azti.es (E.S.); jferrer@azti.es (J.F.)

**Keywords:** omega-3 fatty acid, eicosapentaenoic acid (EPA), *Vibrio* sp., polyunsaturated fatty acid synthase *Pfa*, polyketide synthase (PKS), marine gamma-Proteobacteria

## Abstract

Omega-3 long-chain polyunsaturated fatty acids (LC-PUFAs), such as eicosapentaenoic acid (EPA) (20:5n-3) and docosahexaenoic acid (DHA) (22:6n-3), are considered essential for human health. Microorganisms are the primary producers of omega-3 fatty acids in marine ecosystems, representing a sustainable source of these lipids, as an alternative to the fish industry. Some marine bacteria can produce LC-PUFAs de novo via the Polyunsaturated Fatty Acid (*Pfa)* synthase/ Polyketide Synthase (PKS) pathway, which does not require desaturation and elongation of saturated fatty acids. Cultivation-independent surveys have revealed that the diversity of microorganisms harboring a molecular marker of the *pfa* gene cluster (i.e., *pfa*A-KS domain) is high and their potential distribution in marine systems is widespread, from surface seawater to sediments. However, the isolation of PUFA producers from marine waters has been typically restricted to deep or cold environments. Here, we report a phenotypic and genotypic screening for the identification of omega-3 fatty acid producers in free-living bacterial strains isolated from 5, 500, and 1000 m deep coastal seawater from the Bay of Biscay (Spain). We further measured EPA production in pelagic *Vibrio* sp. strains collected at the three different depths. *Vibrio* sp. EPA-producers and non-producers were simultaneously isolated from the same water samples and shared a high percentage of identity in their 16S rRNA genes, supporting the view that the *pfa* gene cluster can be horizontally transferred. Within a cluster of EPA-producers, we found intraspecific variation in the levels of EPA synthesis for isolates harboring different genetic variants of the *pfa*A-KS domain. The maximum production of EPA was found in a *Vibrio* sp. strain isolated from a 1000 m depth (average 4.29% ± 1.07 of total fatty acids at 10 °C, without any optimization of culturing conditions).

## 1. Introduction

Omega-3 long-chain polyunsaturated fatty acids (LC-PUFAs), such as eicosapentaenoic acid (EPA) (20:5n-3) and docosahexaenoic acid (DHA) (22:6n-3), have a key physiological role in human metabolism [1]. In mammals, omega-3 LC-PUFAs can only be synthesized from the dietary precursor α-linolenic acid (ALA) [2,3], and thus, they are considered essential nutrients. Omega-3 LC-PUFAs are necessary for normal prenatal and child development and, nowadays, their health benefits are well-recognized as they are associated with the improvement of the immune system, neuromuscular function, and neuropsychiatric disorders, among others [1,4,5,6]. The Food and Agriculture Organization has recommended a minimum daily intake of 250 mg of omega-3 LC-PUFAs and up to 300 mg in pregnant women due to their critical biological functions in fetal development [7]. The consumer awareness of their health implications has triggered an exponential increase of the global market demand of omega-3 fatty acids with therapeutic objectives [8]. Besides the fish and seafood incorporated in the diet, fish oil supplements are the predominant commercial source of omega-3 fatty acids as some wild fish families (e.g., Scombridae, Clupeidae, and Salmonidae) consume and accumulate omega-3 fatty acids in their tissues [9]. However, due to the severe concerns associated with wild fisheries, such as environmental damage of fish stocks and bioaccumulation of pollutants, new sustainable sources of omega-3 fatty acids are needed to meet the growing demand of food and nutraceutical industries [2]. 

Microorganisms (bacteria and phytoplankton) are the primary producers of omega-3 PUFAs in marine ecosystems and they transfer these valuable fatty acids to the rest of the food chain. Bacteria also represent a green source of LC-PUFAs, with cheaper downstream purification processes compared with fish oil [9,10,11,12,13]. In the last years, several marine bacterial cultures have been isolated with the ability to produce omega-3 fatty acids and the metabolic pathways behind their synthesis have been identified. Most of the known microbial producers are gamma-Proteobacteria affiliated with the genera *Moritella, Photobacteria, Shewanella, Colwellia, Psychromonas*, and *Vibrio* [14]. In the Flavobacteria class, some isolates affiliated with *Flexibacter* and *Psychroserpens* have also shown the ability to produce omega-3 LC-PUFAs [12]. These isolates have been predominantly retrieved from polar regions and deep ocean habitats characterized by high pressure and/or low temperature [10,12,15] or as part of the gut microbiota of some marine and freshwater fishes [16,17]. A variety of physiological roles of LC-PUFAs in microorganisms have been proposed, including adaptation to psychrophile and piezophile habitats by enhancing membrane fluidity [18,19,20] and acting as antioxidants [21,22,23,24].

While the classical pathway of omega-3 LC-PUFAs’ production in phytoplankton is through desaturases and elongases in aerobic conditions [25], in marine bacteria, the synthesis of these secondary metabolites is mainly via the anaerobic polyketide synthase (PKS) pathway, mediated by a polyunsaturated fatty acid (*Pfa*) synthase [26]. The gene cluster involved in the latter pathway was first deciphered in *Shewanella* sp. strain SCRC-2738 by functionally characterizing a 38 Kbp genomic fragment containing the operon responsible for EPA synthesis [27]. This gene cluster is composed of at least five genes located sequentially (*pfa*A-E), functioning as a multi-enzyme for the de novo synthesis of omega-3 LC-PUFAs [26,28]. Interestingly, the same gene cluster is found in eukaryotes and bacteria, suggesting that these genes can be transferred horizontally across both domains [29]. Since the discovery of the *pfa* gene cluster, several strategies to enhance omega-3 production have been developed, such as using genetic engineering techniques for heterologous production of EPA/DHA in recombinant strains, and the optimization of their culturing conditions [12]. However, the number of isolated bacteria with the ability to produce omega-3 LC-PUFAs remains rather low. Yet, cultivation-independent surveys based on a functional marker of the *pfa* gene cluster (the β-ketoacyl synthase/KS domain from the *pfa*A gene) have revealed that the diversity of these genes in marine bacteria is large, and their distribution in oceanic samples is much more widespread than initially thought [30]. These results suggest that the potential to retrieve omega-3 LC-PUFAs producers from seawater has been, so far, underexplored.

Here, we performed a bioprospection of bacterial producers of omega-3 LC-PUFAs in the Bay of Biscay, a region located in the north-east Atlantic Ocean characterized by high fish and shellfish diversity [31]. Both physiological and molecular analyses were conducted in order to identify new bacterial producers of omega-3 LC-PUFAs in coastal seawater and explore the possible role of these fatty acids in their adaptation to varying temperature conditions.

## 2. Results

### 2.1. Phenotypic and Phylogenetic Analysis of Marine Isolates

From 217 marine isolates obtained from coastal seawater of the Bay of Biscay, ca. 36% were obtained from shallow waters (5 m depth), while 64% were originated from samples collected at 500 and 1000 m depths. No significant variations in viable and culturable bacteria were detected according to the depth of the samples, with a viability range from 2 to 448 CFU/mL (data not shown). All isolated free-living marine bacteria were Gram-negative and catalase positive. Growth was observed at 4, 10, and 25 °C, and therefore the isolates can be considered as psychrotolerant mesophiles. Five different phenotypes were identified based on the colony morphology when grown at 10 °C. Partial 16S rRNA sequences were successfully amplified in 203 strains. The isolates were affiliated with three genera of gamma-Proteobacteria commonly found in marine seawater: *Alteromonas*, *Pseudoalteromonas*, and *Vibrio* sp [32]. Phylotypes closely related to already described species of each genus were found (>99% identity based on partial 16S rRNA genes), although some isolates affiliated with *Alteromonas* and *Pseudoalteromonas* had <99% identity when compared to existant isolates (e.g., *Alteromonas* sp. 611 and 821 and *Pseudoalteromonas* sp. 318) (Figure S1). The relative abundance of each genus varied with depth (Figure S2). *Pseudoalteromonas* sp. was the predominant phylotype in shallow (5 m) and the deepest (1000 m) water samples, while *Vibrio* and *Alteromonas* sp. were mostly found at the 500 m depth.

### 2.2. Phenotypic Screening of LC-PUFA Producers by the TTC Test and GC-FID Lipid Analysis

In order to identify potential LC-PUFAs producers, a first phenotype-based screening was performed based on TTC plates (supplemented with 2,3,5-triphenyl-tetrazolium chloride). About 64% positive-red color colonies were found among the purified isolates and were thus considered as potential LC-PUFAs producers. Similar percentages of TTC-positive colonies were detected at different isolation depths (ca. 22%, 25%, and 17% in 5, 500 and 1000 m depths, respectively). Most TTC-positive colonies were affiliated with *Pseudoalteromonas* sp. at 5 and 1000 m depths, and with *Alteromonas* sp. at the 500 m depth (Figure S2). Although isolates from the three genera showed >99% of identity among them (based on partial 16S rRNA genes) (data not shown), TTC phenotypic variations were observed. Based on this initial screening, we selected 20 TTC-positive isolates from the three genera and depth of isolation and two TTC-negative isolates for GC-FID analysis of their fatty acid profiles. The most abundant fatty acid in the lipid profiles within the assayed isolates was 16:1, followed by 16:0 and 18:1 (Figure S3). All TTC-positive isolates that produced significant amounts of omega-3 (>1% total fatty acids—TFA) at 10 °C, specifically EPA (20:5n-3), were affiliated with *Vibrio* sp. 

### 2.3. Molecular Screening of Omega-3 Fatty Acid Producers by the pfaA-KS Functional Marker and GC-MS Lipid Analysis of Vibrio sp. Isolates 

**Figure 1:**
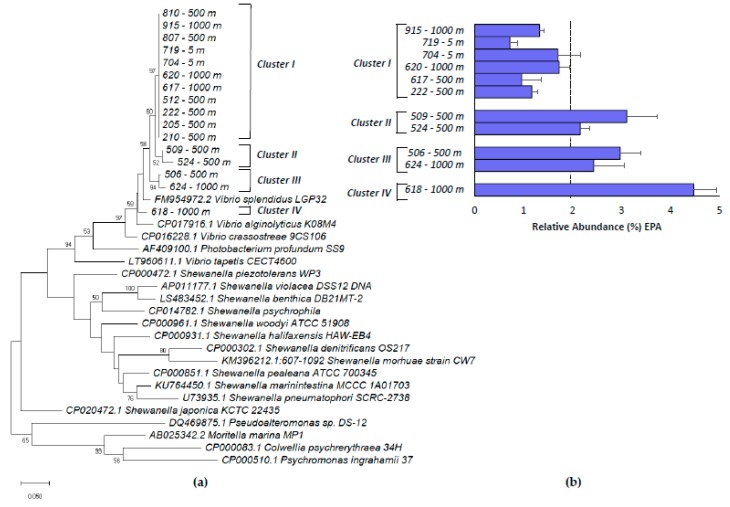
(**a**) Phylogenetic tree of *pfa*A-KS nucleotide sequences. GenBank sequences are identified with the accession numbers and *Vibrio* sp. strains isolated in this study are identified by a number and the depth of isolation in the water column, (**b**) relative abundance of eicosapentaenoic acid (EPA, % of total fatty acids) of representative *Vibrio* sp. isolates in the clusters identified in the phylogenetic tree.

A molecular screening based on the presence of the *pfa*A-KS gene domain was performed on 86 TTC-positive isolates. A positive amplification of the *pfa*A-KS region (ca. 400 bp) was found in 63% TTC-positive *Vibrio* sp. isolates whereas none of the TTC-positive *Pseudoalteromonas* and *Alteromonas* sp. were positive for the *pfa*A-KS amplification (Table S1). While the amino acid sequence of the *pfa*A-KS amplified region was identical in all *pfa*A-KS-positive strains (Figure S4), four clusters were identified (I-IV) at the nucleotide level. The same genetic variant of the *pfa*A-KS was shared among strains isolated at different depths (Figure 1). In order to confirm EPA production in *Vibrio* sp. isolates according to this molecular screening, we re-analyzed by GC-MS a total of 11 *pfa*A-KS positive and 5 *pfa*A-KS negative strains, as representatives from the three water depths of isolation (Figure S5, Figure S6). EPA production was confirmed in all *pfa*A-KS-positive strains and absent in negative strains. Interestingly, different *pfa*A-KS genetic variants identified at the nucleotide level produced different levels of EPA when grown at 10 °C (Figure 1). Specifically, the strains affiliated with cluster I produced a lower quantity of EPA (<2% of total fatty acids) as compared to the other clusters (Student’s T-test, *p* < 0.005). The most promising candidate for EPA production, *Vibrio* sp. 618, displayed a genetic variant of *pfa*A-KS phylogenetically close to that of *Vibrio splendidus* LGP32, with a reported maximum of 2.94% of eicosenoid fatty acids (∑20:1n-9 + 20:2 + 20:4n-6 + 20:5n-3) at 25 °C in previous studies [33] and *Photobacterium profundum* SS9, a well-known EPA producer in psychrophilic conditions (7% of EPA at 4 °C) [28]. Other *Vibrio* sp. harboring the *pfa*A-KS cluster (*V. splendidus* LGP32, *V. alginolyticus* K08M4, *V. crassostreae* 9CS106, and *V. tapetis* CECT4600) were identified from the NCBI Genbank database (see the materials and methods, Section 4.3) and included in the phylogenetic tree, although their ability to produce omega-3 LC-PUFAs has not been determined yet experimentally.

### 2.4. Phylogenetic Analysis of Vibrio Isolates

**Figure 2:**
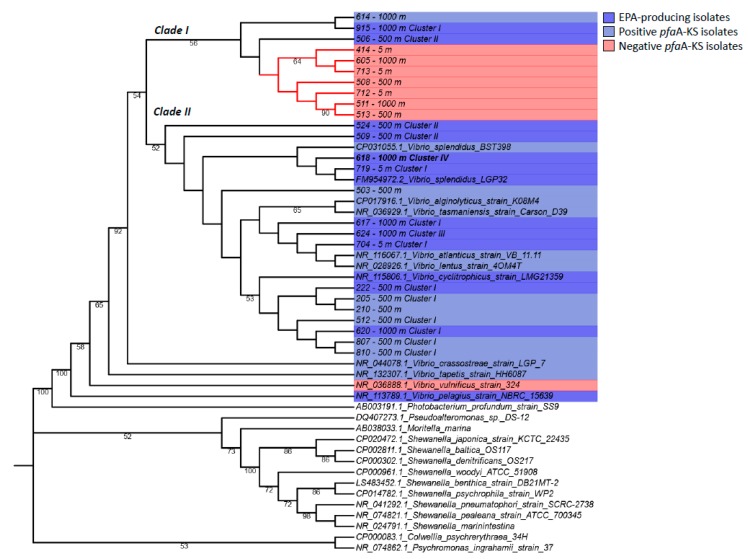
Phylogenetic tree based on 16S rRNA nucleotide sequences. Experimentally demonstrated omega-3 LC-PUFA producers are highlighted in dark blue. Positive *pfa*A-KS isolates are highlighted in light blue and *pfa*A-KS negative isolates in red. Uncolored strains are described gamma-proteobacteria omega-3 producers. GenBank sequences are identified with their correspondence accession numbers. New *Vibrio* sp. isolates are identified by an ID number and depth of isolation (m) in the water column. The best EPA-producer isolate (*Vibrio* sp. 618) is highlighted in bold.

In order to conduct a more robust phylogenetic analysis of Vibrio isolates, an analysis of full-length 16S rRNA genes was carried out (Figure 2). Most of the Vibrio sp. isolates were closely related at the phylogenetic level, and two main clades were observed (clade I and clade II, sharing 99.05% identity among them, Figure 2). Both clades included phylotypes retrieved from the three different depths, confirming their presence in the same environments along the water column. Based on 16S rRNA sequence analysis, Vibrio sp. 618 shared a maximum of 99.85% nucleotide identity with sequences deposited in GenBank, with the bioluminescent Vibrio splendidus LGP32 (FM954972.2) being the most closely related. However, it should be noted that bioluminescence was not observed in any of the isolated Vibrio sp. strains under the assayed conditions. Clades I and II contained members harboring the pfaA-KS marker gene (light blue, Figure 2), and some of them were experimentally confirmed EPA producers (dark blue, Figure 2). However, clade I also included Vibrio isolates, which were not positive for the pfaA-KS marker gene. In general, the phylogenetic relationship of most of the Vibrio isolates based on the 16S rRNA gene was not congruent with that observed based on the pfaA-KS marker (Figure 2).

### 2.5. Genomic Sequence Analysis 

**Figure 3:**
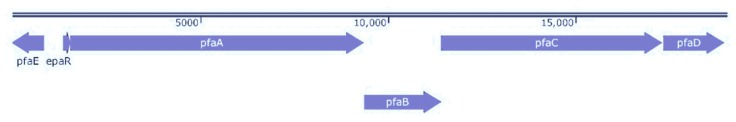
Organization of the genes encoding the EPA biosynthetic polyketide synthase (PKS) cluster present in *Vibrio* sp. 618, as obtained by whole-genome sequencing using the Nanopore MinION platform.

We selected the EPA producer *Vibrio* sp. 618, and the non-producer *Vibrio* sp. 414 for whole genome sequencing. Both strains were taxonomically assigned to *Vibrio tasmaniensis* LGP32 (*Vibrio splendidus* LGP32) by the WIMP Nanopore software (Table S2). The presence of the *pfa*/PKS cluster was confirmed in the genome of *Vibrio* sp. 618, while it was apparently absent in *Vibrio* sp. 414. Based on a BLASTn search, the different *pfa* genes were identified in the assembled *Vibrio* sp. 618 genome. The genetic organization of the EPA biosynthesis cluster in *Vibrio* sp. 618 was composed of *pfa*A–E genes and a hypothetical regulatory gene (*epa*R) (Figure 3). This organization has been previously found in other EPA-producing gamma-proteobacteria strains (e.g., *Shewanella* sp.) and has been designated as a type A *pfa* synthase cluster, in which the order of *pfa* genes is the following: *pfa*A [KS-MAT-ACP(4–6)-KR], *pfa*B [AT], *pfa*C [KS-CLF-DH2], *pfa*D [ER], and *pfa*E [PPTase] [17,29].

### 2.6. Effect of temperature on fatty acid profiles of Vibrio sp. EPA-producers and non-producers 

**Figure 4:**
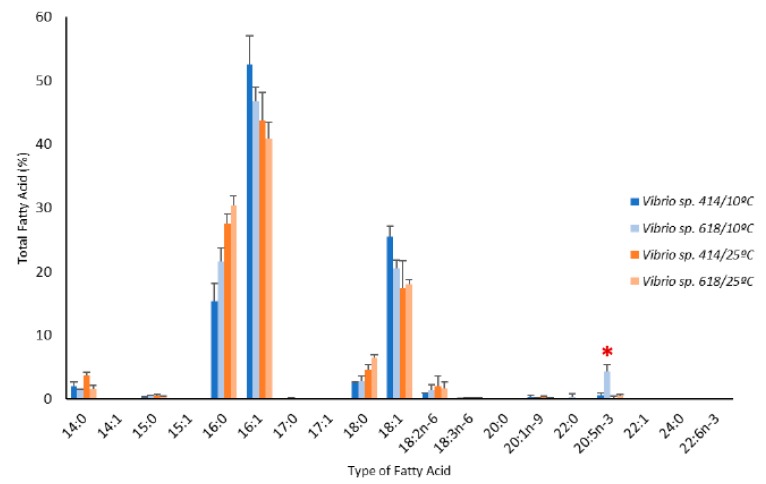
Fatty acid profiles of an EPA producer (*Vibrio* sp. 618) and a non-producer (*Vibrio* sp. 414) grown at 10 and 25 *°*C. The EPA peak has been highlighted with an asterisk.

We tested the effect of temperature on the fatty acid profiles of an EPA-producer isolate (*Vibrio* sp. 618) and a non-producer isolate (*Vibrio* sp. 414). A clear increment in the percentage of monounsaturated fatty acids (MUFAs 16:1 and 18:1) was observed for both strains under cold temperature, particularly in the case of 16:1 (Figure 4). EPA production (>1% TFA) was observed in *Vibrio* sp. 618 at 10 °C but not at 25 °C (Figure 4 and Figure S7). The average production at 10 °C in the latter strain was 1.82 mg/g of dry weight (4.29% of TFA ± 1.07%) after 24 h and up to 2.34 mg/g (7.33% of TFA) after 120 h.

**Figure 5:**
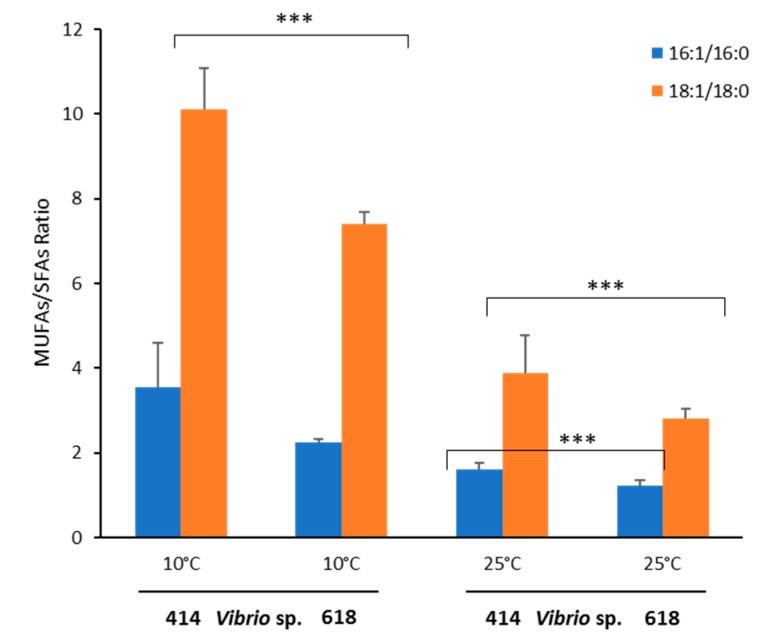
Monounsaturated fatty acids (MUFAs)/saturated fatty acids (SFAs) ratio in *Vibrio* sp. 618 and 414 grown at 10 and 25 °C. (***) indicates statistical significance (Student t-test, *p* < 0.005).

The MUFA/SFA ratio was higher in *Vibrio* sp. 414 (non-producer strain) than in *Vibrio* sp. 618 (EPA producer strain) at both temperatures, especially at 10 °C. Accordingly, the unsaturated index values (see the materials and methods for a definition) observed at 10 °C (91% for EPA producer and 82% for the non-producer strain) were significantly higher than the values obtained at 25 °C (64% for the EPA producer and 68% for the non-producer strain, Student’s t test, *p* < 0.005, Figure 5). Additionally, we performed a PCR-screening to verify the presence of DES9, a key enzyme involved in MUFA (16:1n-9 and 18:1n-9) synthesis by the desaturation of SFAs (16:0 and 18:0). Both *Vibrio* sp. strains were positive for ∆9-des amplification (Table S1).

Finally, the growth rate of both strains at 10 and 25 °C was compared (Figure S8). In mesophilic conditions (25 °C), both strains showed a short lag phase, and the maximal growth rates (µmax) were 0.507 and 0.309 h^−1^ for *Vibrio* sp. 618 and 414, respectively. In psychrophilic conditions (10 °C), the non-producer strain showed a substantially longer lag phase (5.8 h) than the EPA producer strain (Figure S8). However, maximal growth rates were similar in both cases (0.179 and 0.151 h^−1^ for *Vibrio* sp. 618 and 414, respectively) and they reached a similar maximal abundance after 45 h of incubation.

## 3. Discussion

Extreme environments, such as polar waters and the deep ocean, have been the main sources of bacterial producers of omega-3 fatty acids [13,34]. Due to their important role in the fluidity and functioning of cell membranes, LC-PUFAs are presumably key for the adaptation of these microorganisms to cold temperature and high atmospheric pressure conditions [10,19,35,36]. However, a recent survey targeting a molecular marker of the microbial PKS pathway of the synthesis of LC-PUFAs (*pfa*A-KS) in different marine samples has suggested a much more widespread distribution of potential EPA/DHA producers in the ocean [29,30]. Unexpectedly, the maximum relative abundance of potentially EPA-producing bacteria in the latter study was found in surface waters (3 m depth) at 17 °C, in a sample collected off the Scripps Pier (La Jolla, California, CA, USA) [30]. These results suggest that bacterial EPA producers can be potentially isolated from shallow seawater, which would represent a much less challenging source for bioprospection as compared to deep or polar waters. 

In this work, we successfully isolated novel pelagic omega-3 fatty acid producers from surface coastal waters (5 m depth), affiliated with *Vibrio* sp. EPA/DHA-producing *Vibrio* sp. had been previously isolated from deep-sea sediments [14,37,38], marine fish larvae, and freshwater fish gut [16,39]. Although it should be noted that the proportion of EPA-producing *Vibrio* sp. was higher in isolates recovered at 500 and 1000 m depths, here we confirm that bacterial producers of omega-3 LC-PUFAs are not restricted to piezophilic and psychrophilic environments [17,40,41]. 

The plate-based TTC method has been recently proposed for the screening of the production of EPA in Gram-negative bacteria, where positive LC-PUFAs colonies are red colored [42]. In accordance with previous screening studies [17,34,43], we observed that this method causes false positives, as many TTC-positive isolates did not produce EPA/DHA, as confirmed by subsequent GC-FID and GC-MS analyses. In 2016, Dailey and co-workers included for the first time a PCR step for evaluating the presence of the *pfa*A-KS domain in some TTC-positive colonies with previously designed primers [17,30]. The amplification of the *pfa*A-KS domain was experimentally demonstrated in Gram-negative EPA-producing isolates in the latter work, and more recently, in Gram-positive EPA-producing strains [44]. Here, we included a PCR screening step and further measured their lipid content by GC-MS in order to experimentally confirm that *pfa*A-KS positive strains were indeed omega-3 LC-PUFA producers. Based on our results, we strongly recommend the use of *pfa*A-KS PCR screening when analyzing a large number of samples in order to minimize the effort required for GC analysis. This molecular approach has the advantage of being independent of bacterial growth, temperature, and media composition, which are the main sources of variations in the analysis of microbial fatty acid profiles [45,46,47,48]. 

Furthermore, among EPA-producing *Vibrio* sp. strains, we found that different genetic variants of *pfa*A-KS produced different levels of EPA. The isolate *Vibrio* sp. 618, which showed the highest levels of production, harbored a *pfa*A-KS genetic variant similar to that of the isolate *Photobacterium profundum* SS9, which produces a similar quantity of EPA (8.4% of TFA) [49,50], and also belongs to the *Vibrio*naceae family. These results may suggest that the *pfa*A-KS molecular marker might be used as a tool not only for identifying EPA/DHA-producing strains but also for determining genetic variants with different levels of LC-PUFAs production. However, more research should be conducted to confirm this point by increasing the number of isolates tested, and by ensuring a straightforward comparison under similar growth conditions. Additionally, based on the presence of the *pfa* gene cluster in the genomes of some other *Vibrio* isolates (e.g., *V. alginolyticus* strain K08M4, *V. crassostreae* strain LGP7, *V. lentus* strain 4OM4T, *V. splendidus* BST398, *V. tapetis* strain HH6087, *V. tasmaniensis* strain Carson D39), we suggest that they are also potential EPA producers, even if there is no experimental evidence in the literature.

From an environmental perspective, it is interesting that *pfa*A-KS-positive/EPA-producing and *pfa*A-KS-negative/EPA-non-producing *Vibrio* sp. strains, in some cases sharing >99% identity at the phylogenetic level, were isolated simultaneously in the same marine samples. The presence of the complete *pfa* gene cluster (*pfa*A–E) was further confirmed by whole genome sequencing in the strain *Vibrio sp*. 618. The same cluster organization has also been annotated in the genome of *Vibrio splendidus* LG32 [51], the closest strain to the *Vibrio* sp. isolates found in this study. Moreover, the *pfa* gene cluster has been identified in three other *Vibrio* genomes (*Vibrio* sp. MED222, *V. splendidus* 12B01, and *V. splendidus* LGP32), while it was absent in other *Vibrio*nales. These results support the view that the *pfa* cluster can be transferred horizontally among *Vibrio* species, as suggested for other taxa [29,30]. Acquiring these genes may provide a selective advantage in terms of responding to changes in environmental conditions, such as decreases in temperature. In order to explore this hypothesis, we tested the effect of temperature on the growth and fatty acid composition of an EPA-producing and a non-producing isolate (*Vibrio* sp. 618 and 414, respectively), which had a similar fatty acid profile under mesophilic conditions (Figure 4). As reported previously for other *Vibrio* isolates [52,53,54], the major fatty acids of these *Vibrio* strains were 16:1, 16:0, and 18:1. We found that the relative proportion of these fatty acids was temperature dependent for both *Vibrio* sp. 414 and 618. When grown in low temperature conditions (10 *°*C), a higher proportion of MUFAs was found in *Vibrio* sp. 414 as compared to *Vibrio* sp. 618, while in the latter, EPA production was observed. These results suggest that omega-3 LC-PUFAs are not essential for the adaptation of *Vibrio* sp. to cold environments in accordance with previous observations [14,18]. Specifically, the EPA non-producer *Vibrio* sp. strain 414 adapted to low temperature conditions by increasing the ratio of 16:1 and 18:1 to maintain membrane fluidity as it has been described previously in other Vibrionales, such as *Photobacterium profundum* SS9 [18]. Fatty acid desaturases encoded by the ∆9-*des* gene have been demonstrated to be involved in the production of MUFAs 16:1(n-9) and 18:1(n-9) in aerobic conditions [19] in some gamma-proteobacteria, such as *Psychrobacter urativorans*, *Pseudoalteromonas* sp. MLY15, and the Antarctic strain *Pseudomonas* sp. A3 [18,55,56]. Here, the ∆9-*des* gene was amplified in all *Vibrio* sp. isolates, suggesting its potential role for the adaptation of cell membranes to fluctuating environmental conditions in this genus [57]. Finally, although both *Vibrio* sp. strains were able to grow at 10 *°*C, the EPA non-producer strain *Vibrio* sp. 414 showed a longer lag phase compared with *Vibrio* sp. 618, which suggests a faster adaptation to low temperature conditions for the EPA-producing strain. In order to verify the role of the omega-3 fatty acids in these strains, more detailed studies should be performed, for example, generating *Vibrio* sp. *pfa* mutants, or by comparing more EPA producer and non-producer isolates. 

In summary, in this work, we successfully isolated bacterial producers of EPA from shallow coastal seawater affiliated with *Vibrio* sp., confirming the potential of retrieving novel marine omega-3 producers from the surface ocean. Given the easy access to this environmental niche as compared to the deep ocean or polar environments, this represents an important advantage in terms of bioprospection. Regarding the biotechnological potential of the isolated strains, the maximum yield of EPA production in the most promising candidate (*Vibrio* sp. 618 isolated at the 1000 m depth) was 7.33% TFA at 120 h, grown at 10 *°*C without optimization. This value is similar to that observed for *V. cyclotrophicus* (10% TFA) and *V. pelagius* (8.7% TFA) [12], and it remains to be seen if further optimization could substantially increase this yield. We also highlight the great value of using the *pfa*A-KS molecular marker in order to optimize the screening of potential EPA/DHA producers, and its potential to differentiate clusters with different production yields, which needs to be evaluated in future studies.

## 4. Materials and Methods

### 4.1. Isolation and Bioprospection of Omega-3 Fatty Acid Bacterial Isolates and Culture Conditions

Seawater samples were obtained in May 2016 during the Oceanographic cruise TRIENAL in the Bay of Biscay (Spain) onboard the research vessel Ramón Margalef. Samples were collected using a rosette sampler at three coastal stations, from different depths in the same water column: Shallow (5 m), mesopelagic (500 m), and bathypelagic (1,000 m) waters. Seawater samples were stored at 4 °C before isolation, which was close to the environmental temperature in situ at 500 and 1000 m depths; seawater at 5 m depth was approximately 16 °C. Bacteria isolation was performed by plating out the seawater (0.1 mL) in Marine Artificial Seawater (MASW) agar plates containing 25.5 g of Instant Ocean^®^ sea salt, 5 g peptone, 1 g yeast extract, and 15 g of bacteriological agar per liter. The agar plates were incubated in aerobic conditions for 3 days at 10 °C. Isolated colonies (217) were selected based on the phenotypic heterogenicity, streaked over MASW medium and incubated for 5 days at 10 °C for purification and conservation for further analysis. 

### 4.2. TTC Screening of Omega-3 Fatty Acid-Producing Bacteria 

Previously to the TTC screening, gram reaction of the marine isolates was tested by the KOH method [58]. The catalase test was performed using 3% hydrogen peroxide and the oxidase test was carried out using Oxidase Strips (Microgen Camberley, UK). Gram-negative isolates were then plated on MASW agar supplemented with 0.1% (*w*/*v*) 2,3,5-Triphenyl-Tetrazolium chloride (TTC) (Alfa Aesar, Tewksbury, USA) for 3 days at 10 °C for an initial screening of omega-3 producing bacteria, as previously described [42]. TTC-positive (129) and TTC-negative (2) pure isolates were selected for subsequent work. 

### 4.3. Phylogenetic Analysis of the Isolates Based on the 16S rRNA Gene Marker

Amplification of 16S rDNA partial genes of the isolates was performed by colony PCR using the following universal primers (5’-GGTGGAGCATGTGGTTTAATTCGA-3’ and 5’-CCCGGGAACGTATTCACCG-3’) for an initial taxonomic identification of the isolates. Based on this preliminary analysis, the full-length 16S rDNA of those isolates affiliated with *Vibrio* sp. were amplified using the universal primers 27F: 5’-AGAGGTTGATCMTGGCTCAG-3’ and 1525R: 5’-AAGGAG GTGWTCCARCC-3’ (Lane et al., 1991). PCR amplifications were performed using a C1000 Touch Thermal Cycler (Bio-Rad, Watford, UK) and the 2× PCR Master Mix (Thermo Scientific, Waltham, USA) in a total volume of 50 µL under the following conditions: Initial denaturation at 95 *°*C for 3 min, followed by 30 cycles of 94 *°*C for 30s, 50 *°*C for 30 s, and 72 *°*C for 2 min, and a final extension at 72 *°*C for 10 min. ExoSAP-IT™ PCR Product Cleanup Reagent (Thermo Scientific) was used after DNA agarose gel purification of PCR products. DNA sequencing was carried out by STAB VIDA sequencing service (Caparica, Portugal) using 16S-DNA and 27F/1525R primers for obtaining partial and complete sequences according to the Sanger sequencing service’s instructions. The software Bioedit [59] was used for quality assessment of the sequences. BLASTn [60] against the non-redundant nucleotide sequence and the 16S ribosomal RNA sequences (Bacteria and Archaea) databases from NCBI (https://blast.ncbi.nlm.nih.gov/Blast.cgi) were performed for taxonomic assignment. 16S rDNA sequences of the nearest strains and some outgroups were obtained from the NCBI database. For the phylogenetic analysis of *Vibrio* sp. isolates, a phylogenetic tree was built using the complete 16S rDNA sequences with those from other marine gamma-Proteobacteria strains experimentally characterized as omega-3 fatty acid producers, such as *Shewanella* (*S. benthica* ATCC 43992, *S. frigidimarina* ACAM 591, *S. baltica* MAC1, *S. violacea* DSS12, *S. halifaxensis* HAW-EB4, *S. japonica* KMM 3299, *S. piezotolerans* WP3, *S. psychrophile* WP2, *S. pneumatohori* SCRC-2738), *Colwellia* (*C. psychrerythraea* ACAM 550), *Moritella* (*M. marina* MP-1), *Psychromonas* (*P. marina* JCM 10501), *Photobacterium* (*P. profundum* SS9), *Vibrio* (*V. cyclitrophicus*, *V. pelagius,* and *V. splendidus* LGP32). A local BLASTn and BLASTx of *pfa* genes was performed against the NCBI database for searching orthologs of the *pfa*A gene. Strains with *pfa*A sequence hits with *e*-value ≤ 1×10^−30^) were analyzed also for the presence of *pfa* (B–E) genes. The complete predicted PKS genetic cluster (*pfa*A–E) was identified in *S. amazonensis* SB2B, *S. marinintestina*, *S. woodyi* ATTCC 51908, *P. ingrahamii* strain 37, *V. tapetis strain* HH6087, *V. crassostreae* strain LGP7, *V. splendidus* BST398, *V. tasmaniensis* strain Carson D39, *V. atlanticus* strain VB 11.11, *V. alginolyticus* strain K08M4, and *V. lentus* strain 4OM4T, and added to the phylogenetic tree. The 16S rDNA of *V. vulnificus* strain 324 was also added as a PKS-negative strain. All sequences were first assembled and aligned using the MUSCLE algorithm. Phylogenetic distance trees were inferred by maximun likehood analysis using MEGA X software [61]. Confidence was assessed using 1000 replicates’ bootstrapping. The bootstrap 50% cut-off is indicated in the branches. The phylogenetic tree was edited using iTOL [62]. ClustalO was used for pairwise alignment and identity calculation [63].

### 4.4. Molecular Screening of the pfa Cluster 

The β-ketosynthase (KS) domain present in the *pfa*A gene was used as a molecular marker of the PKS pathway by using the degenerated primers *pfa*A-KS [30]. PCR conditions were the following: Initial denaturation at 95 *°*C 3 min, then 30 cycles of 30 s at 95 *°*C, 30 s at 53 *°*C, 60 s at 72 *°*C, and a final extension 10 min at 72 *°*C. KS nucleotide sequences were aligned using MUSCLE, and a maximum likelihood phylogenetic tree was constructed using MEGA X software [61]. *pfa*A-KS sequences of other *Vibrio* species available in GenBank, such as *Vibrio alginolyticus* KM08M4, *V. crassotreae* 9CS106, and *V. tapetis* CECT4600, were also included, even if it has not been experimentally demonstrated that they are omega-3 producers. *Pfa*A-KS amino acid sequences were aligned using Clustal O [63]. Furthermore, delta 9-fatty acid desaturase (∆9-*des*) gene was used as a molecular marker of the aerobic fatty acid desaturation process by using previously designed degenerated primers DE1-DE2 designed for identification of the ∆9-*des* gene in the gamma-proteobacteria *Psychrobacter urativorans* DSM14009 [64]. 

### 4.5. Nucleotide Sequence Accession Numbers

Complete sequences of 16S rDNA and *pfa*A-KS from *Vibrio* sp. isolates were submitted to GenBank under the following accession numbers: MN974023 to MN974047 and MN991210 to MN991224, respectively.

### 4.6. Screening of LC-PUFAs Production in Bacterial Isolates 

A group of 24 strains isolated from 5,500 and 1000 m of depth were selected for lipid analysis. These strains included 22 TTC-positive red isolates affiliated with *Pseudoalteromonas* sp. (10), *Alteromonas* sp. (5), and *Vibrio* sp. (7) and TTC-negative isolates (2). Marine bacterial cultures were grown in 150 mL of MASW medium for 24 h at 10 *°*C in aerobic conditions and 130 rpm. Exponential phase cultures (OD600nm 0.2–0.6) were harvested by centrifugation at 4000 rpm during 20 min. Total lipid extraction of freeze-dried bacterial lyophilized pellet (100–1000 mg) and fatty acid methyl esters (FAMEs) were conducted by following the Bligh and Dyer method (Bligh & Dyer, 1959) with modifications. Fatty acid extractions were achieved adding 3 mL MeOH, 1.5 mL CH_3_Cl, and 1.2 mL water and vortexing for 1 min for homogenization using an IKA™ Ultra-Turrax™ T 10 dispenser (IKA, Staufen, Germany). The same proportions of CH_3_Cl and water were added until the final proportion of the solvents was 3 mL MeOH, 3 mL CH_3_Cl, and 2.4 mL distillated water. Vortexing was repeated for 1 min for re-extraction and tubes were centrifuged 5 min at 3000 rpm. After discarding the upper and intermediate phases, the lower phase was collected. Humidity and insoluble parts were removed from the lower phase by a column (Pasteur pipette) containing NaSO_4_ and glass wool. The extracted fatty acids were methylated by evaporation of the CH_3_Cl layer with N_2_ to dryness and 0.2% (*w*/*v*) NaOCH_3_ and pumice stone were added. Samples were boiled with a reflux condenser for 10 min. Two drops of phenolphthalein were added to the chilled samples and HCl-MeOH (2:1) until they reached complete acidification. Samples were boiled again in the same conditions, and when cooled, 5 mL of n-hexane was added, and tubes were agitated for 1 min. A concentrated solution of NaCl was added to increase the rising volume of the n-hexane-phase, allowing the complete recovery of FAMEs. FAMEs were dried and dissolved in a final volume of 200–1000 µL of *n*-hexane. The Bligh and Dyer method was used for quantification of EPA in *Vibrio* sp. 414 and 618 at two different temperatures (10 and 25 *°*C) in 24 and 120-h cultures. Nonadecanoic acid (C19:0) (19 mg/L) was used as an internal standard (IS) for fatty acid abundance determination (mg/mL). FAMEs were identified with chromatographic standards. FA composition and EPA quantification were performed on an Agilent 5890A model gas chromatograph (GC) equipped with a capillary Agilent D-B 23 p. No 122-2362, J&W Scientific (60 m × 0.25 mm × 0.25 µm) and flame ionization detection (FID) using helium as a carrier gas. The unsaturation index was calculated as the sum of the mean percentages (by weight) of the UFA species multiplied by the number of double bonds [10]. 

### 4.7. Analysis of Lipid Content in Vibrio sp. Strains 

Due to the low content of EPA observed in some *Vibrio* sp. strains by GC-FID, lipid analyses were performed also using FAMEs identification and quantification by gas chromatography/mass spectrometry (GC/MS). Strains were grown at 10 °C on MBSW medium 24 h at 130 rpm. *E. coli* BL21-AI (Invitrogen, ThermoFisher Scientific, Waltham, MA, USA) was included as a negative control. Bacterial cultures were grown in 150 mL of MASW broth 24 h at 10 °C in aerobic conditions and 130 rpm whereas *E. coli* was grown in LB medium. Mid-late exponential phase cells (OD600nm 0.4–0.8) were harvested by centrifugation at 4000 rpm during 20 min. Total cellular fatty acid extraction and FAMEs preparation were carried out following the protocol MIDI–Microbial Identification System, previously described [65] (www.midi-inc.com). All the organic extractions were concentrated by evaporation and dissolved in *n*-hexane (50 µL). GC/MS (Agilent Technologies GC 5975C, autosampler 7683B coupled to MS 7890A) equipped with an Agilent column J&W 112-88A7 (100 m × 250 µm × 0.25 µm) was used with helium as a carrier and usually run at 1.3 mL/min. Data was processed using Agilent ChemStation 7.0 software (Santa Clara, CA, USA). The percentage of total peak area was used for estimating the relative EPA content. The NIST02.L mass spectral library was used for FAMEs confirmation.

### 4.8. Statistical Analysis

Cellular fatty acid composition profiles of bacterial strains were calculated as average ± standard deviation of the same isolation depth. The fatty acid profile for EPA production in *Vibrio* sp. bacterial isolates grown for 24 h was represented as the result of three biological replicates ± standard deviation. Student’s *t*-test were used for calculating significative differences (*p*-value < 0.05) between two samples means (*n* ≥ 3). 

### 4.9. Whole-Genome Sequencing Using the MinION Platform 

The isolates *Vibrio* sp. 414 (isolated at 5 m depth), and 618 (isolated from 1000 m depth) were grown in MB media overnight at 25 °C. Pellet was harvested, and chromosomal DNA was extracted with a QIAamp DNA Mini Kit (QIAGEN) and purified with DNeasy PowerClean Pro Clean Up (QIAGEN, Hilden, Germany). The purified DNA was quantified using a NanoDropTM spectrometer and visualized with an agarose gel. The Qubit dsDNA HS (High Sensitivity) Assay Kit was used for DNA quantification in a Qubit 2.0 fluorometer (Thermo Fisher Scientific). The Nanopore Rapid Sequencing (SQK-RAD004) (Oxford Nanopore Technologies, Oxford, UK) protocol was used for genomic DNA library preparation and sequencing with MinION (Oxford Nanopore Technologies). A flow cell quality control (QC) was performed before loading the library into the device following the manufacturer’s recommendations. The data sets were analyzed by automated MinION Data analysis with EPI2ME Software What’s-In-My-Pot (WIMP, Oxford, UK) workflow. Genome assembly was performed using the SMARTdenovo software. Sequencing data obtained by MinION was deposited in ENA under the study accession number PRJEB36532. BLASTx was used for searching orthologs of *Vibrio tasmaniensis (*LGP32) PKS genes (*pfa*A–E) in both *Vibrio* sp. genomes. SnapGene^®^ Viewer software (GSL Biotech; available at snapgene.com) was used for PKS gene cluster annotation.

### 4.10. Phenotypic Assays of Vibrio sp. Strains

Isolates belonging to the *Vibrio* genus were grown in MASW agar for 48 h at 10 °C and then examined in a dark room at room temperature for visualization of bioluminescence phenotypes. Pre-inocula (1/100) in MASW were used for monitoring the growth of two biological replicates of EPA-producing *Vibrio* sp. 618 and non-producing *Vibrio* sp. 414 in parallel at 10 and 25 °C during 8 h. Bacterial growth was measured spectrophotometrically based on the optical density at 600 nm (OD600nm) along a 45-h incubation at 10 and 25 °C. Growth parameters were calculated using the Roberts and Baranyi complete model incorporated in tthe Fdfit online tool in the ComBase database (combined database on predictive microbiology information) (www.combase.cc) [66]. 

## Supplementary Materials

The following are available online at https://www.mdpi.com/1660-3397/18/2/99/s1, Figure S1: Phylogenetic tree based on partial 16S rRNA nucleotide sequences. *Alteromonas* sp. *Pseudoalteromonas* sp. and *Vibrio* sp. sequences have been incorporated for genus representation. Figure S2: Microbial phylogenetic diversity based on 16S rDNA sequences (shallow water −5 m, 500 m and 1,000 m). Percentage of TTC-positive (+column) and TTC-negative (−column) colonies are shown according to each bacterial genus and seawater depth. Figure S3: Lipid profile of selected TTC-positive isolates grouped by genera. (**a**) *Pseudoalteromonas* sp. isolates. (**b**) *Alteromonas* sp. isolates. (**c**) *Vibrio* sp. isolates. Data obtained by GC-FID analysis following the Blight&Dyer protocol. Figure S4: Multiple-sequence alignment (MSA) of amino acid sequences of amplified *pfa*A-KS domain in *Vibrio* sp. isolates. Isolate number_Depth isolation (meters). WP_012604512.1 (*Vibrio splendidus* LGP32). Figure S5: Lipid profile of selected TTC-positive isolates belonging to *Vibrio* sp. isolates, which were *pfa*A-KS positive (upper panel) and *pfa*A-KS negative (lower panel). Data obtained by GC/MS analysis following the Sasser protocol. Figure S6: GC/MS of 5,8,11,14,17-eicosapentanoic acid methyl ester peak from library (upper panel) compared with one EPA-positive samples (lower panel). Figure S7: GC-FID fatty acid profile of an EPA-producer isolate (*Vibrio* sp. 618, lower pannel) and a non-producer (*Vibrio* sp. 414, upper pannel). Isolates were grown at 10 °C and C19:0 was used as internal standard. Figure S8: Bacterial growth curve at 10 °C/25 °C of EPA-producer *Vibrio* sp. 618 and non-producer *Vibrio* sp. 414. R1 and R2 represent different biological replicates. Table S1: List of phenotypic and genotypic characteristics of gamma-proteobacteria isolates from Biscay Bay. Table S2: Data derived from MinION genome sequencing.
