# Supplementary material for: Novel Vibrio spp. Strains Producing Omega-3 Fatty Acids Isolated from Coastal Seawater
Source: Mar Drugs. 2020 Feb 1;18(2):99. doi: 10.3390/md18020099 (PMC7074563; doi:10.3390/md18020099)
Supplement: Supplementary file 1 [file marinedrugs-18-00099-s001.zip › marinedrugs-697638-supplementary.docx]

**Supplemental Materials**

Novel *Vibrio* spp. Strains Producing Omega-3 Fatty Acids Isolated from Coastal Seawater

Mónica Estupiñán ^1^, Igor Hernández ^2^, Eduardo Saitua ^2^, M. Elisabete Bilbao ^1^, Iñaki Mendibil ^1^, Jorge Ferrer ^2^ and Laura Alonso-Sáez ^1,^*

^1^ AZTI-Tecnalia, Marine Research Unit, Txatxarramendi Irla, 48395 Sukarrieta, Spain; mestupinan@azti.es (M.E.); ebilbao@azti.es(M.E.B.); imendibil@azti.es (I.M.)

^2^ AZTI-Tecnalia Food Research Division, Technology Park of Bizkaia, Astondo Bidea, Building 609, 48160 Derio, Spain; igor.hernandezo@ehu.eus (I.H.); esaitua@azti.es (E.S.); jferrer@azti.es (J.F.)

***** Correspondence: lalonso@azti.es; Tel.:

Figure S1: Phylogenetic tree based on partial 16S rRNA nucleotide sequences. Some *Alteromonas* sp., *Pseudoalteromonas* sp., and *Vibrio* sp. sequences have been incorporated for genus representation. (Attached as an independent PDF file).

Figure S2: Taxonomic composition of isolates based on 16S rDNA sequences from shallow water (5 m), 500 m and 1,000 m depth. The contribution of each genus is shown for all strains, TTC-positive strains (+ column) and TTC-negative (- column) strains isolated at each water depth.


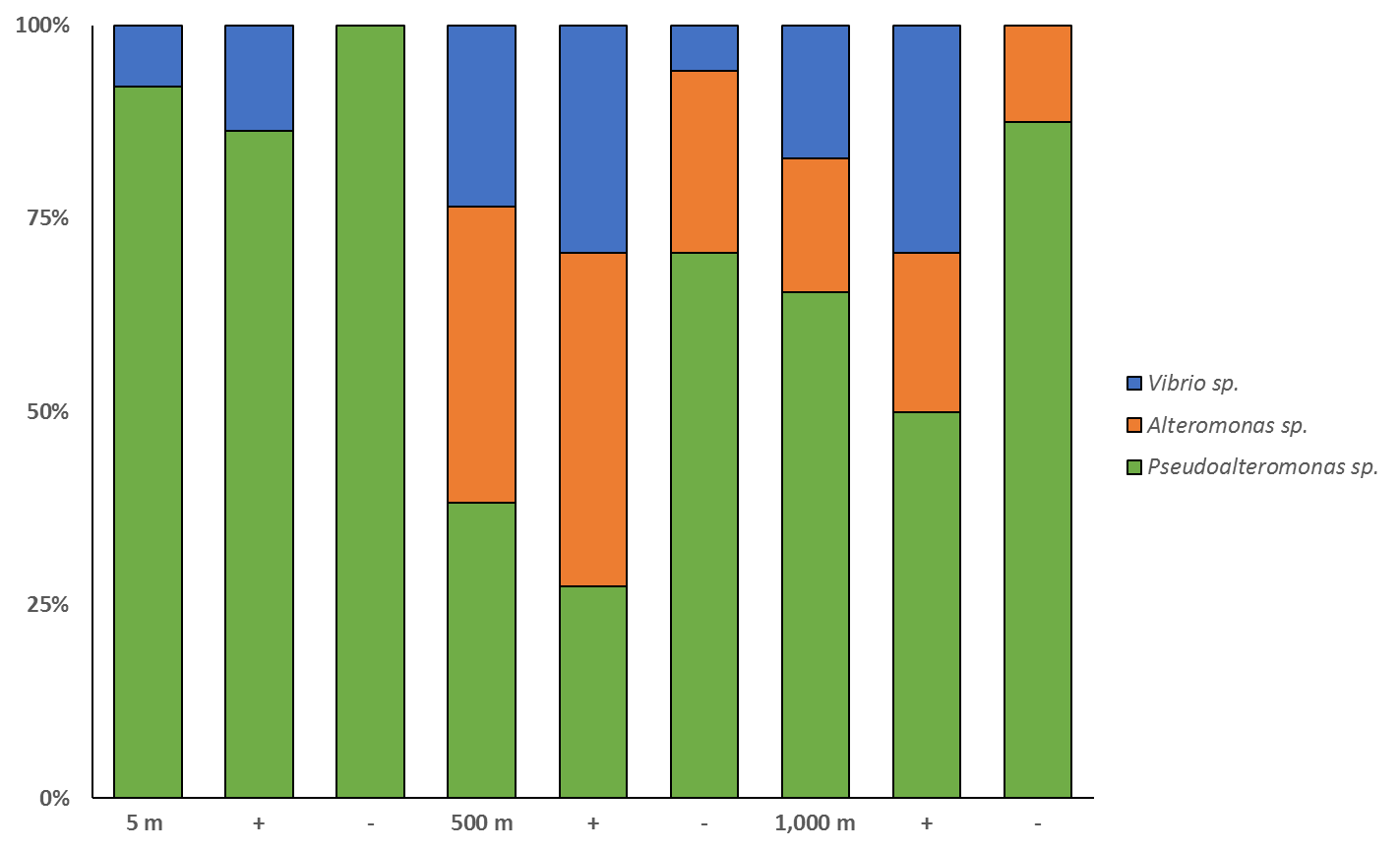


Figure S3: Lipid profile of selected TTC-positive isolates grouped by genera. (**a**) *Pseudoalteromonas* sp. isolates. (**b**) *Alteromonas* sp. isolates. (**c**) *Vibrio* sp. isolates. Data obtained by GC-FID analysis following the Blight&Dyer protocol.


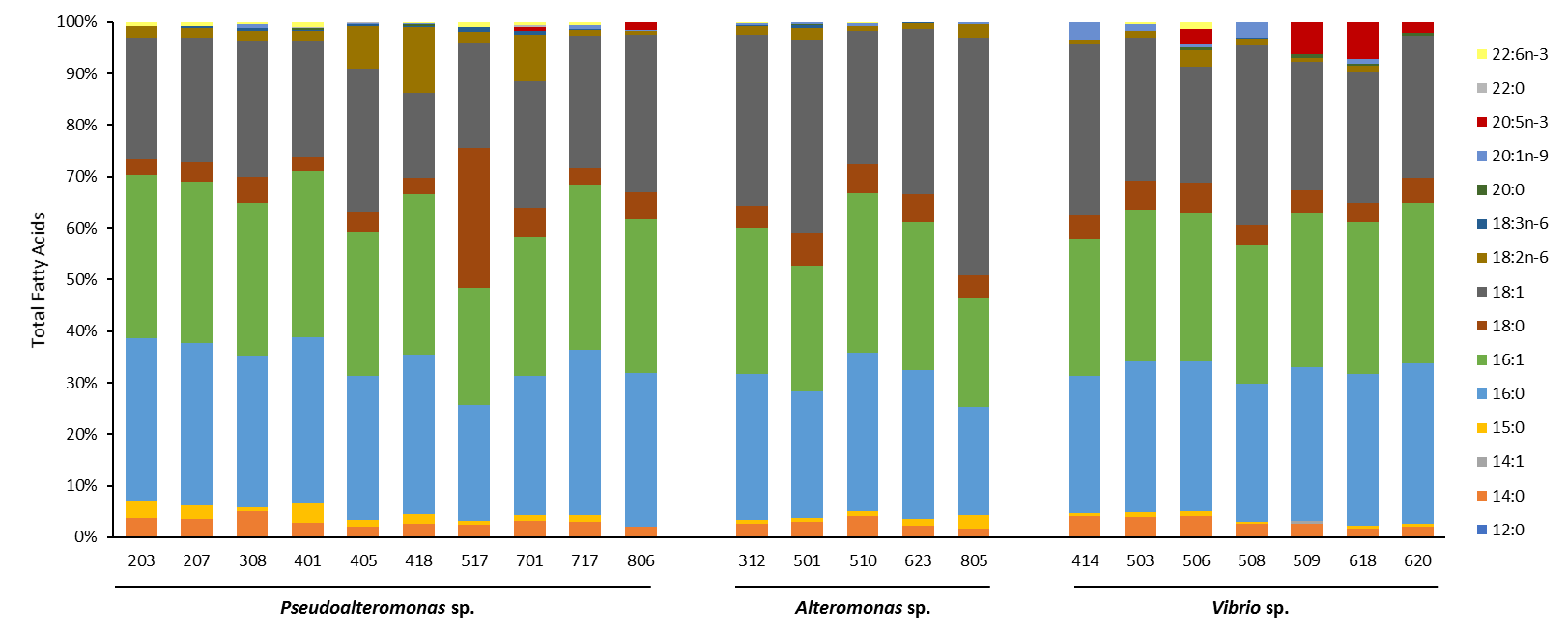


Figure S4: Multiple-sequence alignment (MSA) of amino acid sequences of amplified *pfa*A-KS domain in *Vibrio* sp. isolates. Sequences IDs contain isolate ID number and depth of isolation (meters). WP_012604512.1 (*Vibrio splendidus* LGP32).

Figure S5: Lipid profile of selected TTC-positive isolates belonging to *Vibrio* sp. isolates, which were *pfaA*-KS positive (upper panel) and *pfaA*-KS negative (lower panel). Data obtained by GC/MS analysis following the Sasser protocol.


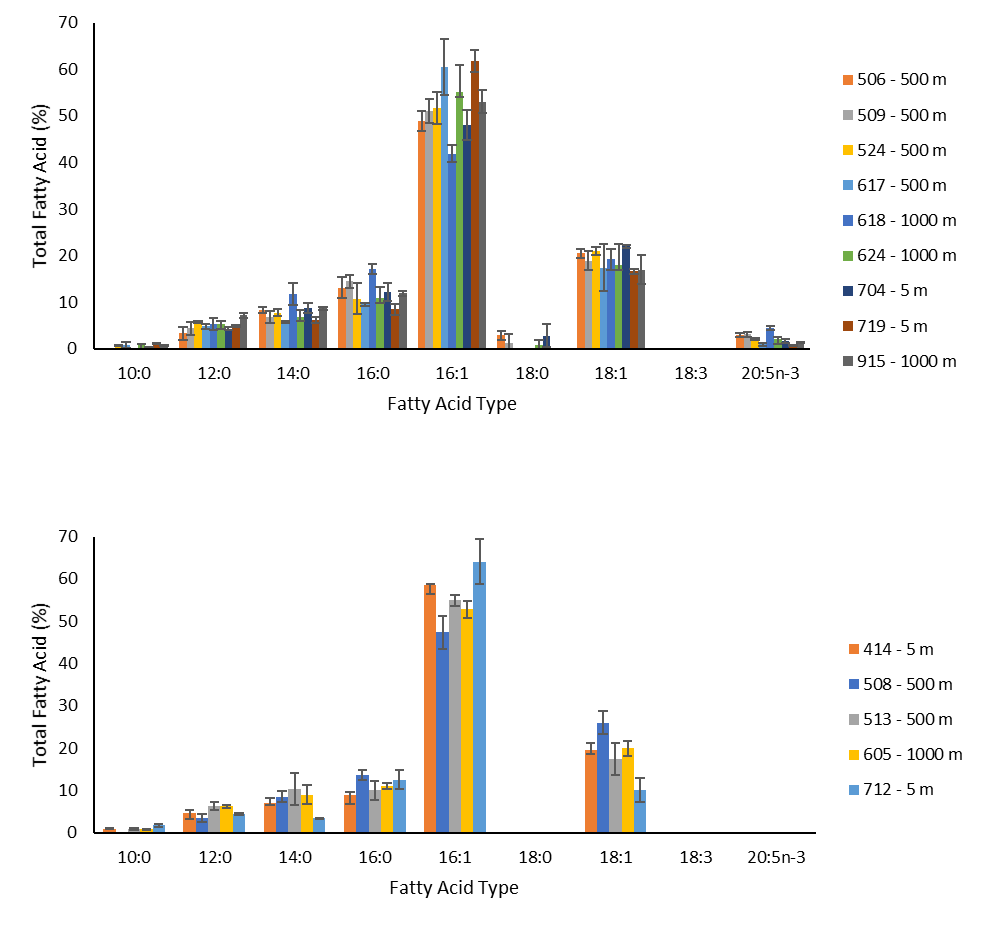


Figure S6. GC/MS of 5,8,11,14,17-eicosapentanoic acid methyl ester peak from library (upper panel) compared with one EPA-positive sample (lower panel).


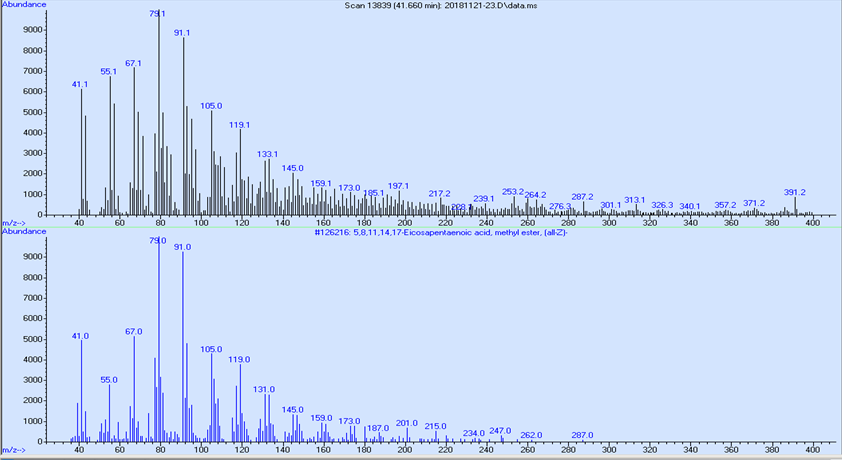


Figure S7: GC-FID fatty acid profile of an EPA-producer isolate (*Vibrio* sp. 618, lower panel) and a non-producer (*Vibrio* sp. 414, upper panel). Isolates were grown at 10 °C and C19:0 was used as internal standard.


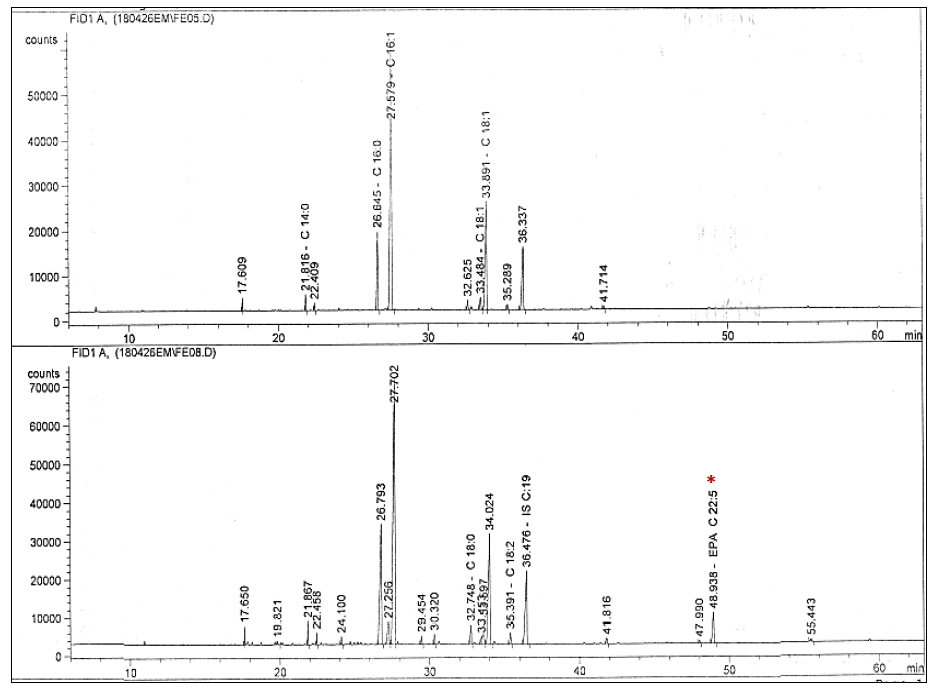


Figure S8. Bacterial growth curve at 10 °C/25 °C of the EPA-producer *Vibrio* sp. 618 and the non-producer *Vibrio* sp. 414. R1 and R2 represent different biological replicates.


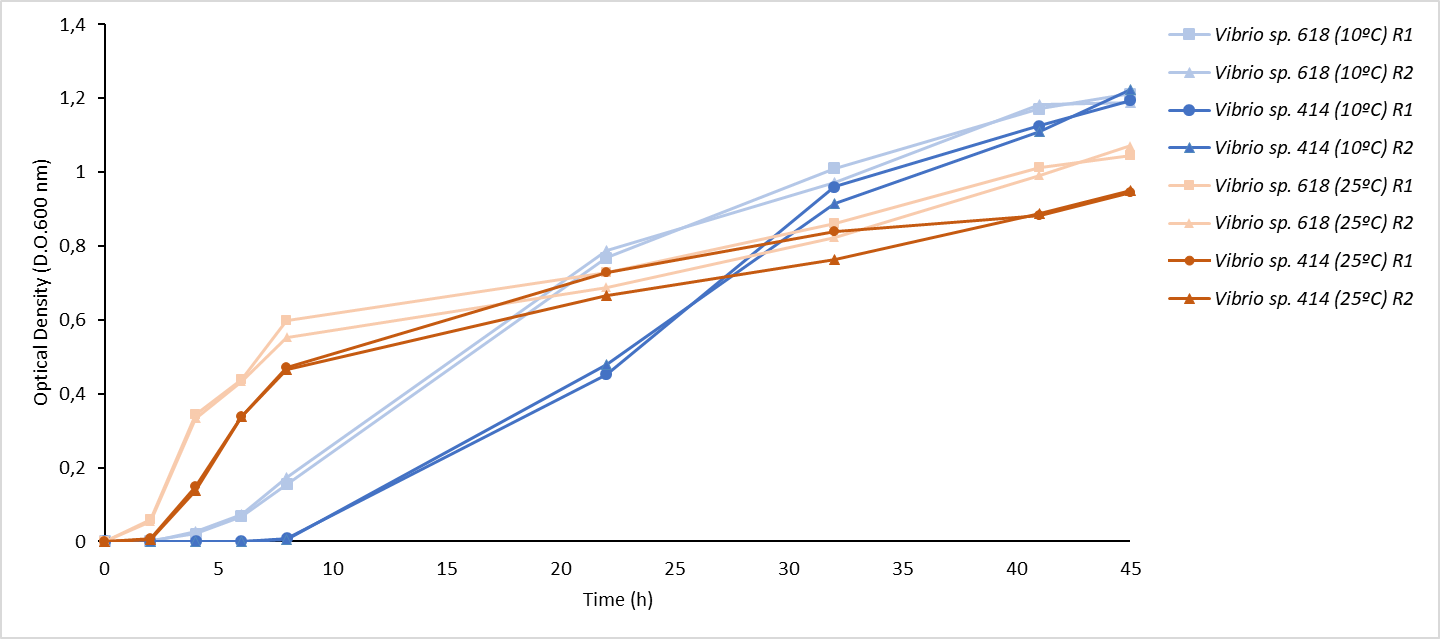


Table S1: List of phenotypic and genotypic characteristics of Gammaproteobacterial isolates from Biscay Bay.

| **Strain Number** | **Depth isolation (m)** | **Genus**  **(16S rRNA)** | **TTC^1^**  **Test** | **EPA^2^ presence** | ***pfa*A-KS amplicon** | **∆9-*des* amplicon** |
| --- | --- | --- | --- | --- | --- | --- |
| 123 | 5 | *Pseudoalteromonas* | + | ND | *-* | ND |
| 203 | 500 | *Pseudoalteromonas* | + | - | *-* | ND |
| 205 | 500 | *Vibrio* | + | ND | *+* | *+* |
| 206 | 500 | *Pseudoalteromonas* | + | ND | *-* | ND |
| 207 | 500 | *Pseudoalteromonas* | + | - | *-* | ND |
| 208 | 500 | *Pseudoalteromonas* | + | ND | *-* | ND |
| 209 | 500 | *Vibrio* | + | ND | *+* | *+* |
| 210 | 500 | *Vibrio* | + | ND | *+* | *+* |
| 215 | 500 | *Alteromonas* | + | ND | - | ND |
| 216 | 500 | *Alteromonas* | + | ND | - | ND |
| 221 | 500 | *Pseudoalteromonas* | + | ND | *-* | ND |
| 222 | 500 | *Vibrio* | + | + | *+* | *+* |
| 308 | 1000 | *Pseudoalteromonas* | + | - | *-* | ND |
| 309 | 1000 | *Pseudoalteromonas* | + | ND | *-* | ND |
| 311 | 1000 | *Pseudoalteromonas* | + | ND | *-* | ND |
| 312 | 1000 | *Alteromonas* | + | - | - | ND |
| 314 | 1000 | *Pseudoalteromonas* | + | ND | *-* | ND |
| 315 | 1000 | *Pseudoalteromonas* | + | ND | *-* | ND |
| 316 | 1000 | *Pseudoalteromonas* | + | ND | *-* | ND |
| 317 | 1000 | *Pseudoalteromonas* | + | ND | *-* | ND |
| 323 | 1000 | *Alteromonas* | + | ND | - | ND |
| 324 | 1000 | *Alteromonas* | + | ND | - | ND |
| 401 | 5 | *Pseudoalteromonas* | + | - | - | ND |
| 405 | 5 | *Pseudoalteromonas* | + | - | - | ND |
| 406 | 5 | *Pseudoalteromonas* | + | ND | - | ND |
| 408 | 5 | *Pseudoalteromonas* | + | ND | - | ND |
| 410 | 5 | *Pseudoalteromonas* | + | ND | - | ND |
| 411 | 5 | *Pseudoalteromonas* | + | ND | - | ND |
| 414 | 5 | *Vibrio* | + | - | *+* | *+* |
| 415 | 5 | *Pseudoalteromonas* | + | - | - | ND |
| 416 | 5 | *Pseudoalteromonas* | - | - | - | ND |
| 417 | 5 | *Pseudoalteromonas* | + | ND | - | ND |
| 418 | 5 | *Pseudoalteromonas* | + | - | - | ND |
| 419 | 5 | *Pseudoalteromonas* | - | ND | ND | ND |
| 420 | 5 | *Pseudoalteromonas* | + | ND | - | ND |
| 421 | 5 | *Pseudoalteromonas* | + | ND | - | ND |
| 424 | 5 | *Pseudoalteromonas* | + | ND | - | ND |
| 501 | 500 | *Alteromonas* | + | - | - | ND |
| 503 | 500 | *Vibrio* | + | + | + | + |
| 504 | 500 | *Alteromonas* | + | ND | - | ND |
| 505 | 500 | *Alteromonas* | + | ND | - | ND |
| 506 | 500 | *Vibrio* | + | + | + | + |
| 507 | 500 | *Alteromonas* | + | ND | - | ND |
| 508 | 500 | *Vibrio* | + | - | - | + |
| 509 | 500 | *Vibrio* | + | + | + | + |
| 510 | 500 | *Alteromonas* | + | - | - | ND |
| 511 | 500 | *Vibrio* | + | + | + | + |
| 512 | 500 | *Vibrio* | + | + | - | + |
| 513 | 500 | *Vibrio* | + | - | - | + |
| 515 | 500 | *Pseudoalteromonas* | + | ND | - | ND |
| 517 | 500 | *Pseudoalteromonas* | + | - | - | - |
| 518 | 500 | *Alteromonas* | + | ND | - | - |
| 524 | 500 | *Vibrio* | + | + | + | + |
| 603 | 1000 | *Pseudoalteromonas* | + | ND | - | ND |
| 605 | 1000 | *Vibrio* | + | - | - | + |
| 611 | 1000 | *Alteromonas* | + | ND | - | ND |
| 613 | 1000 | *Pseudoalteromonas* | + | ND | - | ND |
| 617 | 1000 | *Vibrio* | + | + | + | + |
| 618 | 1000 | *Vibrio* | + | + | + | + |
| 620 | 1000 | *Vibrio* | + | + | + | + |
| 621 | 1000 | *Vibrio* | + | + | + | + |
| 623 | 1000 | *Alteromonas* | + | - | - | ND |
| 624 | 1000 | *Vibrio* | + | + | + | + |
| 701 | 5 | *Pseudoalteromonas* | + | - | - | ND |
| 702 | 5 | *Pseudoalteromonas* | + | ND | - | ND |
| 704 | 5 | *Vibrio* | + | + | + | + |
| 707 | 5 | *Pseudoalteromonas* | + | ND | - | ND |
| 709 | 5 | *Pseudoalteromonas* | + | ND | - | ND |
| 712 | 5 | *Vibrio* | + | - | - | + |
| 713 | 5 | *Vibrio* | + | - | - | + |
| 714 | 5 | *Pseudoalteromonas* | + | - | - | ND |
| 715 | 5 | *Pseudoalteromonas* | + | ND | - | ND |
| 717 | 5 | *Pseudoalteromonas* | + | - | - | ND |
| 718 | 5 | *Pseudoalteromonas* | + | ND | - | ND |
| 719 | 5 | *Vibrio* | + | + | + | + |
| 721 | 5 | *Pseudoalteromonas* | + | ND | - | ND |
| 722 | 5 | *Pseudoalteromonas* | + | ND | - | ND |
| 724 | 5 | *Pseudoalteromonas* | + | ND | - | ND |
| 726 | 5 | *Pseudoalteromonas* | + | ND | - | ND |
| 803 | 500 | *Alteromonas* | + | - | - | ND |
| 804 | 500 | *Alteromonas* | + | ND | - | ND |
| 805 | 500 | *Alteromonas* | + | - | - | ND |
| 807 | 500 | *Vibrio* | + | + | + | + |
| 808 | 500 | *Alteromonas* | + | ND | - | ND |
| 810 | 500 | *Vibrio* | + | + | + | + |
| 811 | 500 | *Pseudoalteromonas* | + | ND | - | ND |
| 812 | 500 | *Alteromonas* | + | ND | - | ND |
| 813 | 500 | *Alteromonas* | + | ND | - | ND |
| 814 | 500 | *Alteromonas* | + | ND | - | ND |
| 815 | 500 | *Alteromonas* | + | ND | - | ND |
| 816 | 500 | *Alteromonas* | + | ND | - | ND |
| 818 | 500 | *Alteromonas* | + | ND | - | ND |
| 820 | 500 | *Alteromonas* | + | ND | - | ND |
| 821 | 500 | *Alteromonas* | + | ND | - | ND |
| 822 | 500 | *Pseudoalteromonas* | - | ND | - | ND |
| 914 | 1000 | *Alteromonas* | + | ND | - | ND |
| 915 | 1000 | *Vibrio* | + | + | + | + |

^1^ TTC (2,3,5-Triphenyl-Tetrazolim chloride), ^2^EPA (eicosapentaenoic acid, 20:5n-3) >1% of Total Fatty Acid (TFA). ND (non-determined).

**Table S2.** Data derived from MinION genome sequencing.

| **Strain** | ***Vibrio* sp. 414** | ***Vibrio* sp. 618** |
| --- | --- | --- |
| LC50 | 5 | 9 |
| Genome size (Mbp) | 5,8 | 4,8 |
| WIMP^1^ (q-score) | 17,4 | 20,6 |

^1^ What’s-In-My-Pot (ONT) Software.
