# Supplementary figures and images for: Novel Vibrio spp. Strains Producing Omega-3 Fatty Acids Isolated from Coastal Seawater
Source: Mar Drugs. 2020 Feb 1;18(2):99. doi: 10.3390/md18020099 (PMC7074563; doi:10.3390/md18020099)

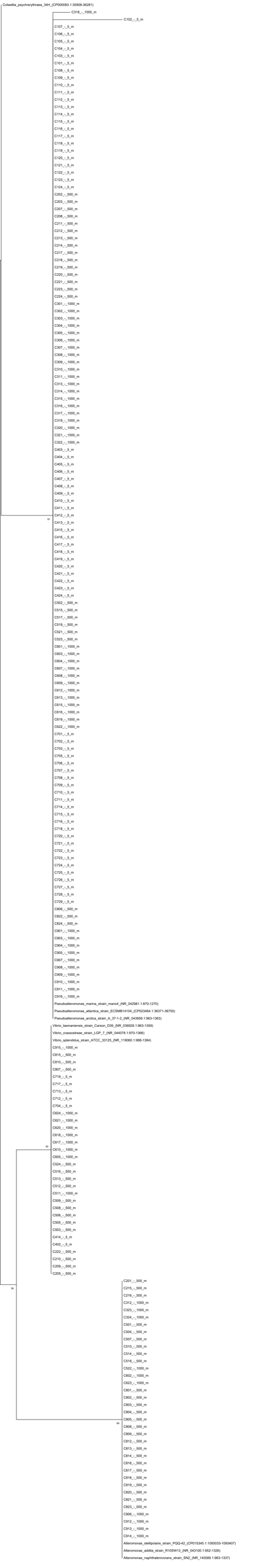

Supplement: Supplementary file 1 [file marinedrugs-18-00099-s001.zip › Figure S1.pdf]
